# The genome sequence of a hoverfly, *Syrphus vitripennis *(Meigen, 1822)

**DOI:** 10.12688/wellcomeopenres.20837.1

**Published:** 2024-02-19

**Authors:** Olga Sivell, Duncan Sivell, Steven Falk, Natural History Museum Genome Acquisition Lab, University of Oxford and Wytham Woods Genome Acquisition Lab, Darwin Tree of Life Barcoding collective, Wellcome Sanger Institute Tree of Life Management, Samples and Laboratory team, Wellcome Sanger Institute Scientific Operations: Sequencing Operations, Wellcome Sanger Institute Tree of Life Core Informatics team, Tree of Life Core Informatics collective, Darwin Tree of Life Consortium

**Affiliations:** 1 Natural History Museum, London, England, UK; 2 Independent researcher, Kenilworth, England, UK

**Keywords:** Syrphus vitripennis, a hoverfly, genome sequence, chromosomal, Diptera

## Abstract

We present a genome assembly from an individual male *Syrphus vitripennis* (a hoverfly; Arthropoda; Insecta; Diptera; Syrphidae). The genome sequence is 388.8 megabases in span. Most of the assembly is scaffolded into 5 chromosomal pseudomolecules, including the XY sex chromosome. The mitochondrial genome has also been assembled and is 18.33 kilobases in length.

## Species taxonomy

Eukaryota; Metazoa; Eumetazoa; Bilateria; Protostomia; Ecdysozoa; Panarthropoda; Arthropoda; Mandibulata; Pancrustacea; Hexapoda; Insecta; Dicondylia; Pterygota; Neoptera; Endopterygota; Diptera; Brachycera; Muscomorpha; Eremoneura; Cyclorrhapha; Aschiza; Syrphoidea; Syrphidae; Syrphinae; Syrphini; *Syrphus*; *Syrphus vitripennis* (Meigen, 1822) (NCBI:txid224256).

## Background

*Syrphus vitripennis* Meigen, 1822 is a medium-sized (body length 8–11 mm, wing length 7–10 mm) yellow and black wasp-mimicking hoverfly. It is one of the five species from the genus *Syrphus* occurring in Britain, three of which are common but difficult to separate. The genus is characterised by the hairs on the upper side of the lower calypter. The males of *S. vitripennis* are indistinguishable from *S. rectus* Osten Sacken, 1875, a North American species, found in Europe (including Britain) on a few occasions ( Ball & Morris, 2015; van Veen, 2014). The females can be separated based on the colour of the hind femur: black in the basal two-thirds in *S. vitripennis* and yellow on the basal half in *S. rectus.* The second basal cell of *S. vitripennis* is only partially covered in microtrichia, which distinguishes this species from *S. ribesii* (Linnaeus, 1758) and *S. torvus* Osten Sacken, 1875 which have their second basal cell entirely covered with microtrichia ( Ball & Morris, 2015; van Veen, 2014).

*Syrphus vitripennis* is a Holarctic species, in North America it occurs from Alaska to California ( Speight, 2017). This species is highly migratory, moving seasonally to higher latitudes in the spring and lower latitudes in autumn. They have been observed in June on the Isles of Scilly and in mainland Cornwall migrating northwards into the rest of Britain ( Hawkes *et al.*, 2022). This species is common and widely distributed in Britain and Ireland, occurring in wide range of lowland habitats including woodland, clearings, gardens, parks and hedgerows. The adults are on the wing from March to November, peaking in July and August ( Ball *et al.*, 2011; Ball & Morris, 2015; Speight, 2017).

The adults feed on flowers while the larvae predate aphids on various trees, shrubs, and tall herbaceous plants such as alder *Alnus*, birch *Betula*, *Prunus*, *Viburnum*, hop *Humulus* and thistles *Cirsium* ( Speight, 2020). The species overwinters as larva ( Kula, 1982). The egg of *Syrphus vitripennis* has been described by Chandler (1968), the larva by Dusek and Laska (1964), and the puparium by Dussaix (2013).

The complete mitochondrial genome of *Syrphus vitripennis* was reported by Liu *et al.* (2020). The high-quality genome of *Syrphus vitripennis* presented here was sequenced as part of the Darwin Tree of Life Project, a collaborative effort to sequence all named eukaryotic species in the Atlantic Archipelago of Britain and Ireland. It will aid research into the taxonomy, biology and phylogeny of the species. A chromosomally complete genome sequence for *Syrphus vitripennis* was based on a male specimen from Luton.

## Genome sequence report

**Figure 1.**
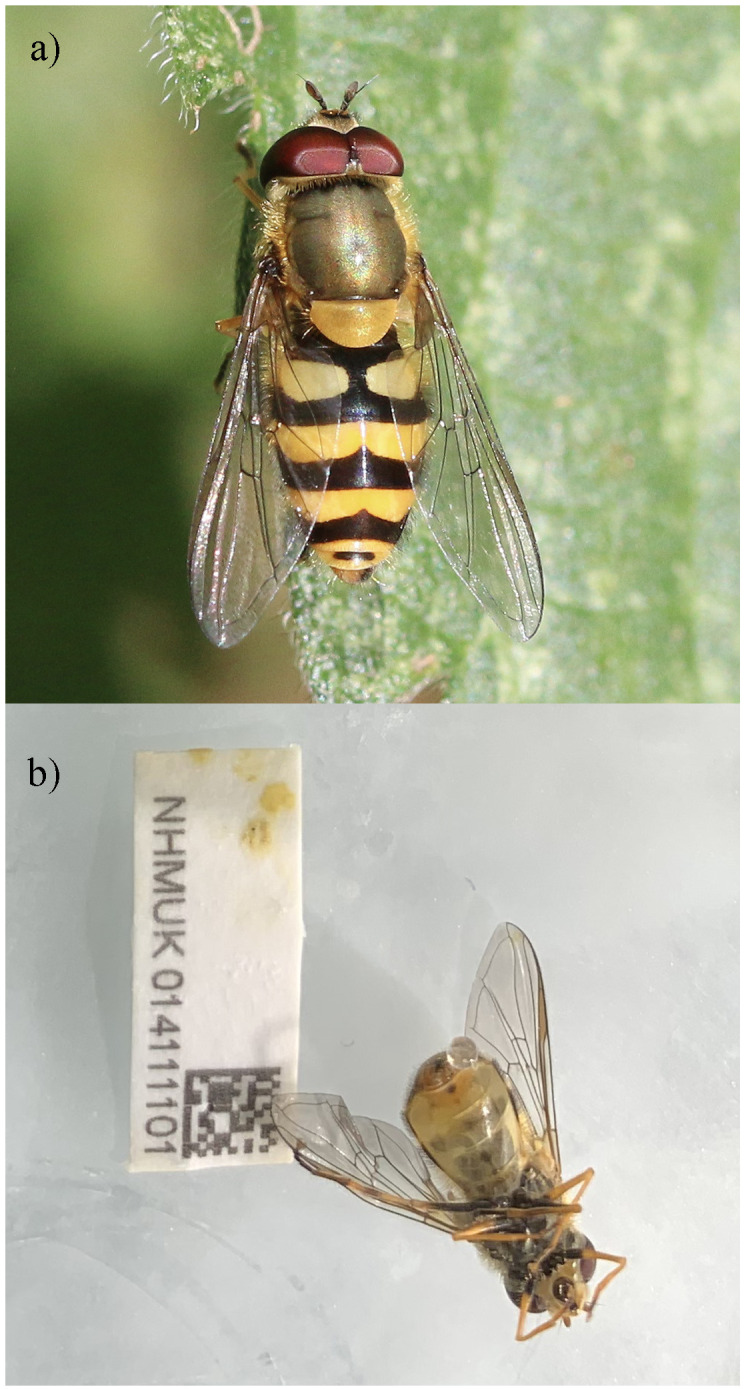
**a**) Hoverfly ( *Syrphus vitripennis*) male, Cumnor Hill Oxford. Photograph by Charles J. Sharpe **b**) Photograph of the *Syrphus vitripennis* (specimen ID NHMUK014111101, idSyrVitr1) specimen used for genome sequencing.

The genome was sequenced from one male *Syrphus vitripennis* ( Figure 1) collected from Wigmore, Luton, England, UK (51.88, –0.37). A total of 45-fold coverage in Pacific Biosciences single-molecule HiFi long reads was generated. Primary assembly contigs were scaffolded with chromosome conformation Hi-C data. Manual assembly curation corrected 158 missing joins or mis-joins and removed one haplotypic duplication, reducing the scaffold number by 18.53%, and increasing the scaffold N50 by 87.19%.

**Figure 2.**
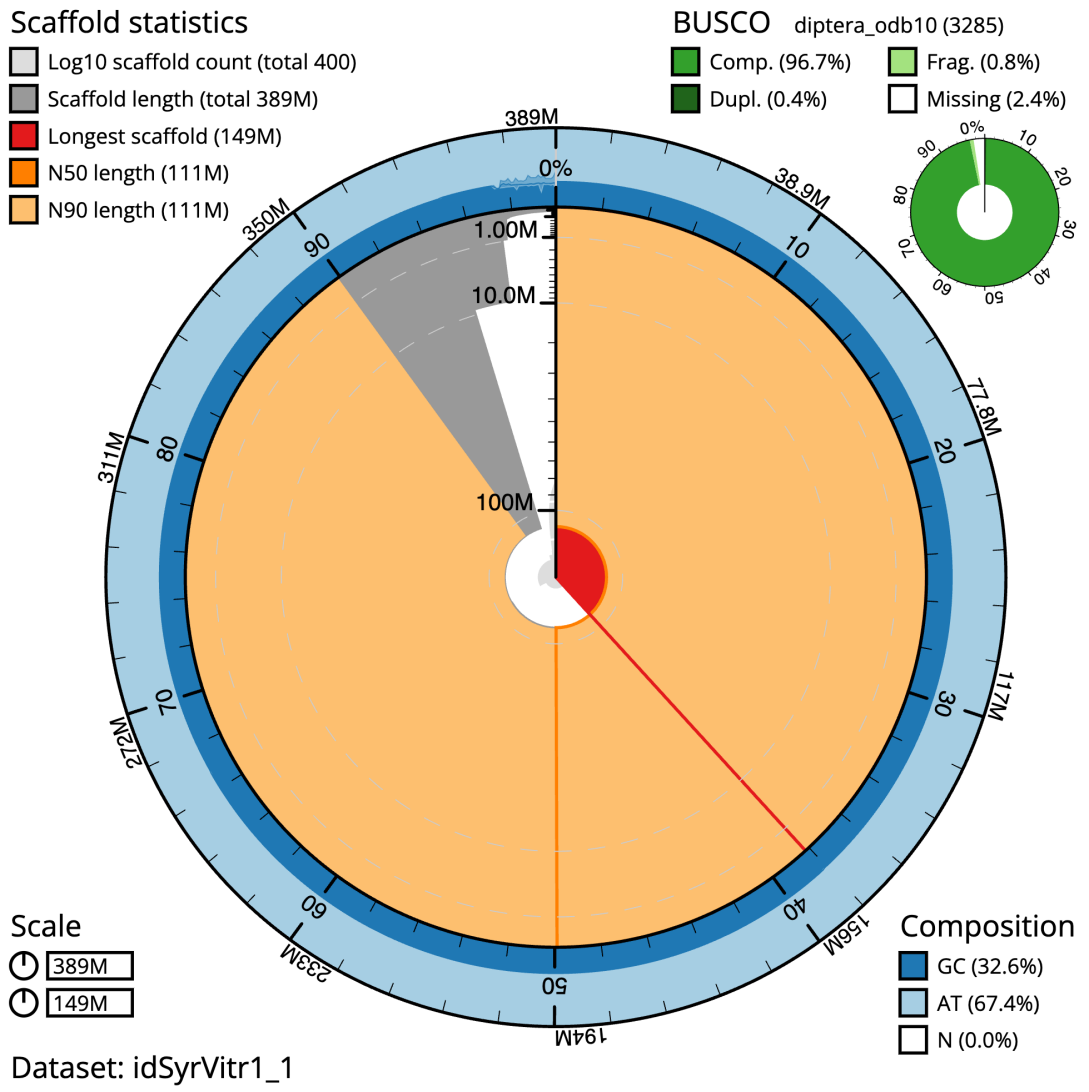
Genome assembly of *Syrphus vitripennis*, idSyrVitr1.1: metrics. The BlobToolKit Snailplot shows N50 metrics and BUSCO gene completeness. The main plot is divided into 1,000 size-ordered bins around the circumference with each bin representing 0.1% of the 388,834,270 bp assembly. The distribution of scaffold lengths is shown in dark grey with the plot radius scaled to the longest scaffold present in the assembly (148,792,233 bp, shown in red). Orange and pale-orange arcs show the N50 and N90 scaffold lengths (110,905,286 and 110,522,704 bp), respectively. The pale grey spiral shows the cumulative scaffold count on a log scale with white scale lines showing successive orders of magnitude. The blue and pale-blue area around the outside of the plot shows the distribution of GC, AT and N percentages in the same bins as the inner plot. A summary of complete, fragmented, duplicated and missing BUSCO genes in the diptera_odb10 set is shown in the top right. An interactive version of this figure is available at https://blobtoolkit.genomehubs.org/view/idSyrVitr1_1/dataset/idSyrVitr1_1/snail.

**Figure 3.**
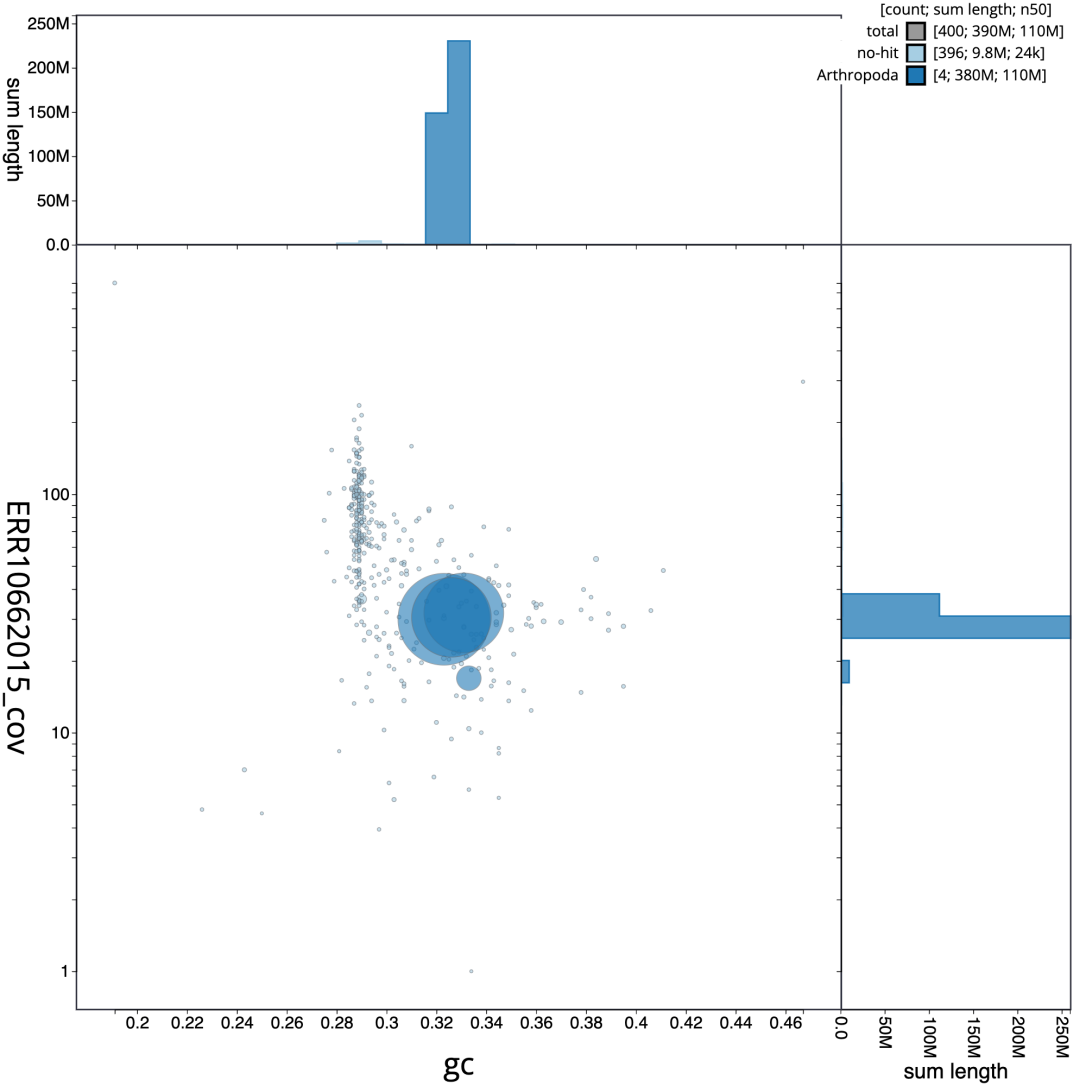
Genome assembly of *Syrphus vitripennis*, idSyrVitr1.1: BlobToolKit GC-coverage plot. Scaffolds are coloured by phylum. Circles are sized in proportion to scaffold length. Histograms show the distribution of scaffold length sum along each axis. An interactive version of this figure is available at https://blobtoolkit.genomehubs.org/view/idSyrVitr1_1/dataset/idSyrVitr1_1/blob.

**Figure 4.**
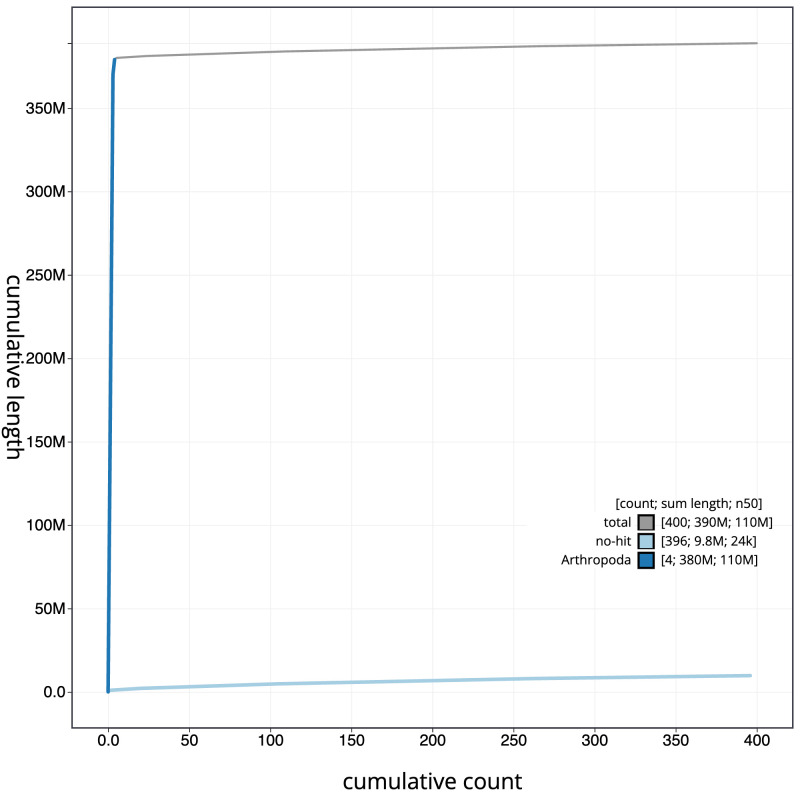
Genome assembly of *Syrphus vitripennis*, idSyrVitr1.1: BlobToolKit cumulative sequence plot. The grey line shows cumulative length for all scaffolds. Coloured lines show cumulative lengths of scaffolds assigned to each phylum using the buscogenes taxrule. An interactive version of this figure is available at https://blobtoolkit.genomehubs.org/view/idSyrVitr1_1/dataset/idSyrVitr1_1/cumulative.

**Figure 5.**
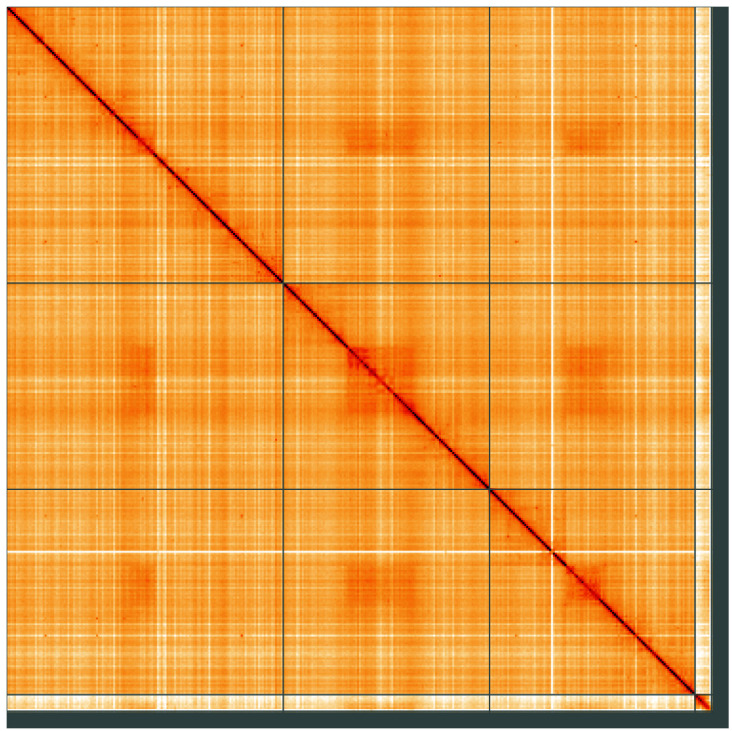
Genome assembly of *Syrphus vitripennis*, idSyrVitr1.1: Hi-C contact map of the idSyrVitr1.1 assembly, visualised using HiGlass. Chromosomes are shown in order of size from left to right and top to bottom. An interactive version of this figure may be viewed at https://genome-note-higlass.tol.sanger.ac.uk/l/?d=DwdApaIqR-eOeeDXHwDppQ.

The final assembly has a total length of 388.8 Mb in 399 sequence scaffolds with a scaffold N50 of 110.9 Mb ( Table 1). The snailplot in Figure 2 provides a summary of the assembly statistics, while the distribution of assembly scaffolds on GC proportion and coverage is shown in Figure 3. The cumulative assembly plot in Figure 4 shows curves for subsets of scaffolds assigned to different phyla. Most (97.72%) of the assembly sequence was assigned to 5 chromosomal-level scaffolds, representing 3 autosomes and the X and Y sex chromosome. Chromosome-scale scaffolds confirmed by the Hi-C data are named in order of size ( Figure 5; Table 2). While not fully phased, the assembly deposited is of one haplotype. Contigs corresponding to the second haplotype have also been deposited. The mitochondrial genome was also assembled and can be found as a contig within the multifasta file of the genome submission.

**Table 1.** Genome data for *Syrphus vitripennis*, idSyrVitr1.1.

Project accession data		
Assembly identifier	idSyrVitr1.1	
Species	*Syrphus vitripennis*	
Specimen	idSyrVitr1	
NCBI taxonomy ID	224256	
BioProject	PRJEB57888	
BioSample ID	SAMEA7521530	
Isolate information	idSyrVitr1, male: thorax (DNA sequencing), head (Hi-C sequencing) idSyrVitr4, male: abdomen (RNA sequencing)	
Assembly metrics *		*Benchmark*
Consensus quality (QV)	56.9	*≥ 50*
*k*-mer completeness	99.99%	*≥ 95%*
BUSCO **	C:96.7%[S:96.4%,D:0.4%], F:0.8%,M:2.4%,n:3,285	*C ≥ 95%*
Percentage of assembly mapped to chromosomes	97.72%	*≥ 95%*
Sex chromosomes	XY	*localised homologous pairs*
Organelles	Mitochondrial genome: 18.33 kb	*complete single alleles*
Raw data accessions		
PacificBiosciences SEQUEL II	ERR10662015	
Hi-C Illumina	ERR10614869	
PolyA RNA-Seq Illumina	ERR11837458	
Genome assembly		
Assembly accession	GCA_958431115.1	
*Accession of alternate haplotype*	GCA_958336355.1	
Span (Mb)	388.8	
Number of contigs	876	
Contig N50 length (Mb)	2.2	
Number of scaffolds	399	
Scaffold N50 length (Mb)	110.9	
Longest scaffold (Mb)	148.79	

* Assembly metric benchmarks are adapted from column VGP-2020 of “Table 1: Proposed standards and metrics for defining genome assembly quality” from ( Rhie *et al.*, 2021). ** BUSCO scores based on the diptera_odb10 BUSCO set using version 5.3.2. C = complete [S = single copy, D = duplicated], F = fragmented, M = missing, n = number of orthologues in comparison. A full set of BUSCO scores is available at https://blobtoolkit.genomehubs.org/view/idSyrVitr1_1/dataset/idSyrVitr1_1/busco.

**Table 2.** Chromosomal pseudomolecules in the genome assembly of *Syrphus vitripennis*, idSyrVitr1.

INSDC accession	Chromosome	Length (Mb)	GC%
OY288108.1	1	148.79	32.5
OY288109.1	2	110.91	33.0
OY288110.1	3	110.52	32.5
OY288111.1	X	8.86	33.5
OY288112.1	Y	0.89	29.0
OY288113.1	MT	0.02	19.0

The estimated Quality Value (QV) of the final assembly is 56.9 with *k*-mer completeness of 99.99%, and the assembly has a BUSCO v5.3.2 completeness of 96.7% (single = 96.4%, duplicated = 0.4%), using the diptera_odb10 reference set ( *n* = 3,285).

Metadata for specimens, barcode results, spectra estimates, sequencing runs, contaminants and pre-curation assembly statistics are given at https://links.tol.sanger.ac.uk/species/224256.

## Methods

### Sample acquisition and nucleic acid extraction

A male *Syrphus vitripennis* (specimen ID NHMUK014111101, ToLID idSyrVitr1) was collected from Wigmore Park, Luton, England, UK (latitude 51.88, longitude –0.37) on 2020-07-07 by netted. The specimen was collected by Olga Sivell (Natural History Museum, London) and identified by Duncan Sivell (Natural History Museum) and preserved on dry ice.

The specimen used for RNA sequencing (specimen ID Ox001241, ToLID idSyrVitr4) was collected from Wytham Woods, Oxfordshire (biological vice-county Berkshire), UK (latitude 51.77, longitude –1.31) on 2021-04-2019. The specimen was collected and identified by Steven Falk (independent researcher) and preserved on dry ice.

The workflow for high molecular weight (HMW) DNA extraction at the Wellcome Sanger Institute (WSI) includes a sequence of core procedures: sample preparation; sample homogenisation, DNA extraction, fragmentation, and clean-up. In sample preparation, the idSyrVitr1 sample was weighed and dissected on dry ice ( Jay *et al.*, 2023). Tissue from the thorax was homogenised using a PowerMasher II tissue disruptor ( Denton *et al.*, 2023a). 

HMW DNA was extracted using the Manual MagAttract v1 protocol ( Strickland *et al.*, 2023b). DNA was sheared into an average fragment size of 12–20 kb in a Megaruptor 3 system with speed setting 30 ( Todorovic *et al.*, 2023). Sheared DNA was purified by solid-phase reversible immobilisation ( Strickland *et al.*, 2023a): in brief, the method employs a 1.8X ratio of AMPure PB beads to sample to eliminate shorter fragments and concentrate the DNA. The concentration of the sheared and purified DNA was assessed using a Nanodrop spectrophotometer and Qubit Fluorometer and Qubit dsDNA High Sensitivity Assay kit. Fragment size distribution was evaluated by running the sample on the FemtoPulse system.

RNA was extracted from abdomen tissue of idSyrVitr4 in the Tree of Life Laboratory at the WSI using the RNA Extraction: Automated MagMax™ *mir*Vana protocol ( do Amaral *et al.*, 2023). The RNA concentration was assessed using a Nanodrop spectrophotometer and a Qubit Fluorometer using the Qubit RNA Broad-Range Assay kit. Analysis of the integrity of the RNA was done using the Agilent RNA 6000 Pico Kit and Eukaryotic Total RNA assay.

Protocols developed by the Tree of Life laboratory are publicly available on protocols.io ( Denton *et al.*, 2023b).

### Sequencing

Pacific Biosciences HiFi circular consensus DNA sequencing libraries were constructed according to the manufacturers’ instructions. Poly(A) RNA-Seq libraries were constructed using the NEB Ultra II RNA Library Prep kit. DNA and RNA sequencing was performed by the Scientific Operations core at the WSI on Pacific Biosciences SEQUEL II (HiFi) and Illumina NovaSeq 6000 (RNA-Seq) instruments. Hi-C data were also generated from head tissue of idSyrVitr1 using the Arima2 kit and sequenced on the Illumina NovaSeq 6000 instrument.

### Genome assembly, curation and evaluation

Assembly was carried out with Hifiasm ( Cheng *et al.*, 2021) and haplotypic duplication was identified and removed with purge_dups ( Guan *et al.*, 2020). The assembly was then scaffolded with Hi-C data ( Rao *et al.*, 2014) using YaHS ( Zhou *et al.*, 2023). The assembly was checked for contamination and corrected as described previously ( Howe *et al.*, 2021). Manual curation was performed using HiGlass ( Kerpedjiev *et al.*, 2018) and Pretext ( Harry, 2022). The mitochondrial genome was assembled using MitoHiFi ( Uliano-Silva *et al.*, 2023), which runs MitoFinder ( Allio *et al.*, 2020) or MITOS ( Bernt *et al.*, 2013) and uses these annotations to select the final mitochondrial contig and to ensure the general quality of the sequence.

A Hi-C map for the final assembly was produced using bwa-mem2 ( Vasimuddin *et al.*, 2019) in the Cooler file format ( Abdennur & Mirny, 2020). To assess the assembly metrics, the *k*-mer completeness and QV consensus quality values were calculated in Merqury ( Rhie *et al.*, 2020). This work was done using Nextflow ( Di Tommaso *et al.*, 2017) DSL2 pipelines “sanger-tol/readmapping” ( Surana *et al.*, 2023a) and “sanger-tol/genomenote” ( Surana *et al.*, 2023b). The genome was analysed within the BlobToolKit environment ( Challis *et al.*, 2020) and BUSCO scores ( Manni *et al.*, 2021; Simão *et al.*, 2015) were calculated.

Table 3 contains a list of relevant software tool versions and sources.

**Table 3.** Software tools: versions and sources.

Software tool	Version	Source
BlobToolKit	4.2.1	https://github.com/blobtoolkit/blobtoolkit
BUSCO	5.3.2	https://gitlab.com/ezlab/busco
Hifiasm	0.16.1-r375	https://github.com/chhylp123/hifiasm
HiGlass	1.11.6	https://github.com/higlass/higlass
Merqury	MerquryFK	https://github.com/thegenemyers/MERQURY.FK
MitoHiFi	2	https://github.com/marcelauliano/MitoHiFi
PretextView	0.2	https://github.com/wtsi-hpag/PretextView
purge_dups	1.2.3	https://github.com/dfguan/purge_dups
sanger-tol/genomenote	v1.0	https://github.com/sanger-tol/genomenote
sanger-tol/readmapping	1.1.0	https://github.com/sanger-tol/readmapping/tree/1.1.0
YaHS	1.2a	https://github.com/c-zhou/yahs

### Wellcome Sanger Institute – Legal and Governance

The materials that have contributed to this genome note have been supplied by a Darwin Tree of Life Partner. The submission of materials by a Darwin Tree of Life Partner is subject to the **‘Darwin Tree of Life Project Sampling Code of Practice’**, which can be found in full on the Darwin Tree of Life website here. By agreeing with and signing up to the Sampling Code of Practice, the Darwin Tree of Life Partner agrees they will meet the legal and ethical requirements and standards set out within this document in respect of all samples acquired for, and supplied to, the Darwin Tree of Life Project. 

Further, the Wellcome Sanger Institute employs a process whereby due diligence is carried out proportionate to the nature of the materials themselves, and the circumstances under which they have been/are to be collected and provided for use. The purpose of this is to address and mitigate any potential legal and/or ethical implications of receipt and use of the materials as part of the research project, and to ensure that in doing so we align with best practice wherever possible. The overarching areas of consideration are:

• Ethical review of provenance and sourcing of the material

• Legality of collection, transfer and use (national and international) 

Each transfer of samples is further undertaken according to a Research Collaboration Agreement or Material Transfer Agreement entered into by the Darwin Tree of Life Partner, Genome Research Limited (operating as the Wellcome Sanger Institute), and in some circumstances other Darwin Tree of Life collaborators.

## Data Availability

European Nucleotide Archive: *Syrphus vitripennis*. Accession number PRJEB57888; https://identifiers.org/ena.embl/PRJEB57888 ( Wellcome Sanger Institute, 2023). The genome sequence is released openly for reuse. The *Syrphus vitripennis* genome sequencing initiative is part of the Darwin Tree of Life (DToL) project. All raw sequence data and the assembly have been deposited in INSDC databases. The genome will be annotated using available RNA-Seq data and presented through the Ensembl pipeline at the European Bioinformatics Institute. Raw data and assembly accession identifiers are reported in Table 1.

## Author information

Members of the University of Oxford and Wytham Woods Genome Acquisition Lab are listed here: https://doi.org/10.5281/zenodo.7125292.

Members of the Natural History Museum Genome Acquisition Lab are listed here: https://doi.org/10.5281/zenodo.7139035.

Members of the Darwin Tree of Life Barcoding collective are listed here: https://doi.org/10.5281/zenodo.4893703.

Members of the Wellcome Sanger Institute Tree of Life Management, Samples and Laboratory team are listed here: https://doi.org/10.5281/zenodo.10066175.

Members of Wellcome Sanger Institute Scientific Operations: Sequencing Operations are listed here: https://doi.org/10.5281/zenodo.10043364.

Members of the Wellcome Sanger Institute Tree of Life Core Informatics team are listed here: https://doi.org/10.5281/zenodo.10066637.

Members of the Tree of Life Core Informatics collective are listed here: https://doi.org/10.5281/zenodo.5013541.

Members of the Darwin Tree of Life Consortium are listed here: https://doi.org/10.5281/zenodo.4783558.
