# Supplementary material for: The genome sequence of a hoverfly, Syrphus vitripennis (Meigen, 1822)
Source: Wellcome Open Res. 2024 Feb 19;9:78. [Version 1] doi: 10.12688/wellcomeopenres.20837.1 (PMC11184275; doi:10.12688/wellcomeopenres.20837.1)
Supplement: Peer review history

# Reviewer report: Reviewer response for version 1

**DOI:** 10.21956/wellcomeopenres.23056.r85255

**Published:** 2024-06-17

**Authors:** Kevin M Moran

**Affiliations:** 1 Biology, The University of Memphis, Memphis, Tennessee, USA

The approach presented in this report on the sequencing and assembly of the genome of *Syrphus vitripennis *is excellent, and I have no doubt that it will be of great value to researchers.

 It is modern, the raw data is high quality, and the methodology is appropriate and understandable. Additionally, the authors have made the raw data and genome publicly available. Based on my own investigation of the data, the resulting genome is high quality and has excellent coverage.

 I appreciate the detail into which the report goes regarding the background of this species as well as the difficulty regarding its identification.

 One idea that might prove helpful is grabbing the COI barcode region and testing it against the BOLD database. Syrphids especially are extremely well sampled and this would serve as an additional check on the species identity of the specimen for difficult groups.

 Additionally, it would be helpful if the literary resource used to determine the identity of this species was provided in the text. Perhaps this was implied somewhere, however, it should be outright stated.

Are sufficient details of methods and materials provided to allow replication by others?

Yes

Is the rationale for creating the dataset(s) clearly described?

Yes

Are the datasets clearly presented in a useable and accessible format?

Yes

Are the protocols appropriate and is the work technically sound?

Yes

Reviewer Expertise:

Phylogenetics, Syrphid Taxonomy, Bioinformatics

I confirm that I have read this submission and believe that I have an appropriate level of expertise to confirm that it is of an acceptable scientific standard.

# Reviewer report: Reviewer response for version 1

**DOI:** 10.21956/wellcomeopenres.23056.r85258

**Published:** 2024-06-11

**Authors:** Marco Gerdol

**Affiliations:** 1 University of Trieste, Trieste, Italy

This manuscript reports the genome assembly of the hoverfly *Syrphus vitripennis.* This genome was sequenced within the Darwin Tree of Life project, and both sequencing and assembly were carried out using well established protocols. Hence, I have no technical concerns. All reported metrics indicate that the quality of this resource was high and in line with expectations.

 As it often happens for the Darwin Tree of Life genomes, however, no gene annotation track was available. Although the authors mentioned that the genome will be annotated by using available RNA-seq resources, an assembled genome lacking annotation is only of partial usefulness for the scientific community, and I would urge the authors to include a link to this important resource as soon as possible. I know for a fact that several other DToL genomes have been annotated using the Ensembl Rapid pipeline. The use of this pipeline would offer the scientific community to have a standardized, albeit imperfect, preliminary gene annotation track.

Are sufficient details of methods and materials provided to allow replication by others?

Yes

Is the rationale for creating the dataset(s) clearly described?

Yes

Are the datasets clearly presented in a useable and accessible format?

Partly

Are the protocols appropriate and is the work technically sound?

Yes

Reviewer Expertise:

Evolutionary geniomics

I confirm that I have read this submission and believe that I have an appropriate level of expertise to confirm that it is of an acceptable scientific standard, however I have significant reservations, as outlined above.

# Reviewer report: Reviewer response for version 1

**DOI:** 10.21956/wellcomeopenres.23056.r85264

**Published:** 2024-06-03

**Authors:** Jerome Hui

**Affiliations:** 1 The Chinese University of Hong Kong, Hong Kong, Hong Kong

Sivell, Sivell, Falk and colleagues report the genome sequence of hoverfly *Syrphus vitripennis *(Meigen, 1822). This species is common and widespread in Britain. Prior to this report, molecular data available for this species are mainly the ribosomal and mitochondrial sequences. This new genome resource will be useful for further studies, such as understanding the cryptic species/subpopulations, ecological, evolutionary, and genomics questions related to insects.

 This genome resource is excellent from the summary statistics, with excellent BUSCO scores, high sequence continuity (scaffold N50), and majority of sequences contained on the 3 pseudochromosomes (plus mitochondrion and sex chromosomes). To sum up, this is a valuable contribution from the Darwin Tree of Life Consortium.

Are sufficient details of methods and materials provided to allow replication by others?

Yes

Is the rationale for creating the dataset(s) clearly described?

Yes

Are the datasets clearly presented in a useable and accessible format?

Yes

Are the protocols appropriate and is the work technically sound?

Yes

Reviewer Expertise:

Genomics, evolution, invertebrates

I confirm that I have read this submission and believe that I have an appropriate level of expertise to confirm that it is of an acceptable scientific standard.
